# Revival of the heterologous prime‐boost technique in COVID‐19: An outlook from the history of outbreaks

**DOI:** 10.1002/hsr2.531

**Published:** 2022-02-23

**Authors:** Amna Siddiqui, Alishba Adnan, Munib Abbas, Shafaq Taseen, Sidhant Ochani, Mohammad Yasir Essar

**Affiliations:** ^1^ Department of MBBS Karachi Medical and Dental College Karachi City Pakistan; ^2^ Department of MBBS Khairpur Medical College Khairpur Mir's Pakistan; ^3^ Department of MBBS Kabul University of Medical Sciences Kabul Afghanistan

**Keywords:** COVID‐19 vaccines, Ebola, hepatitis B, heterologous prime‐boost technique, HIV, influenza virus, malaria, Mix and Match approach, outbreaks, tuberculosis

## Abstract

**Background:**

The heterologous prime‐boost vaccination technique is not novel as it has a history of deployment in previous outbreaks.

**Aim:**

Hence, this narrative review aims to provide critical insight for reviving the heterologous prime‐boost immunization strategy for SARS‐CoV‐2 vaccination relative to a brief positive outlook on the mix‐dose approach deployed in previous and existing outbreaks (ie, Ebola virus disease (EVD), malaria, tuberculosis, hepatitis B, HIV and influenza virus).

**Methodology and Materials:**

A Boolean search was carried out to find the literature in MEDLINE‐PubMed, Clinicaltrials, and Cochrane Central Register of Controlled Trials databases up till December 22, 2021, using the specific keywords that include “SARS‐CoV2”, “COVID‐19”, “Ebola,” “Malaria,” “Tuberculosis,” “Human Immunodeficiency Virus,” “Hepatitis B,” “Influenza,” “Mix and match,” “Heterologous prime‐boost,” with interposition of “OR” and “AND.” Full text of all the related articles in English language with supplementary appendix was retrieved. In addition, the full text of relevant cross‐references was also retrieved.

**Results:**

Therefore, as generally evident by the primary outcomes, that is, safety, reactogenicity, and immunogenicity reported and updated by preclinical and clinical studies for addressing previous and existing outbreaks so far, including COVID‐19, it is understood that heterologous prime‐boost immunization has been proven successful for eliciting a more efficacious immune response as of yet in comparison to the traditional homologous prime‐boost immunization regimen.

**Discussion:**

Accordingly, with increasing cases of COVID‐19, many countries such as Germany, Pakistan, Canada, Thailand, and the United Kingdom have started administering the heterologous vaccination as the technique aids to rationalize the usage of the available vaccines to aid in the global race to vaccinate majority to curb the spread of COVID‐19 efficiently. However, the article emphasizes the need for more large controlled trials considering demographic details of vaccine recipients, which would play an essential role in clearing the mistrust about safety concerns to pace up the acceptance of the technique across the globe.

**Conclusion:**

Consequently, by combatting the back‐to‐back waves of COVID‐19 and other challenging variants of concerns, including Omicron, the discussed approach can also help in addressing the expected evolution of priority outbreaks as coined by WHO, that is, “Disease X” in 2018 with competency, which according to WHO can turn into the “next pandemic” or the “next public health emergency,” thus would eventually lead to eradicating the risk of “population crisis.”

## 1 INTRODUCTION

The outbreak of severe acute respiratory syndrome 2 (SARS‐CoV‐2), started in Hubei province of China at the end of 2019, has led to a cluster of pneumonia cases in Wuhan. The World Health Organization declared the coronavirus disease 2019 (COVID‐19) a global pandemic on March 11, 2020.^1^ The best identified and cost‐effective strategy to prevent infectious diseases is via safe vaccination dosing.^2^ The essential goal of vaccination is to generate potent and long‐term protection against diseases. Thus, to combat the deadly consequence of the COVID‐19 outbreak, scientists accelerated the global vaccine development for SARS‐CoV‐2. Vaccines against COVID‐19 are available in variety and prepared using conventional techniques using inactivated or live attenuated viruses. Other approaches utilize recombinant proteins and virtual vector‐based vaccines.^3^ The concerns regarding traditional vaccination include safety issues, undesirable host reactions, and problematic culturing of dangerous microorganisms that prompted the development of subunit vaccines.^4^, ^5^ However, recombinant subunit vaccines are weakly immunogenic and necessitate additional components to improve protective immunity. Thus, it is recommended to enhance the effectiveness of subunit vaccines through a prime‐boosting immunization strategy.^4^

A prime‐boost vaccination strategy is an immunization program using the identical immunogen during the primary and booster doses (Homologous prime‐boost immunization) or using a different immunogen (Heterologous prime‐boost immunization) to boost the immunogenic priming regimen.^6^ The majority of existing immunization regimens incorporate a second homologous booster dose after a month of the prime‐dosing regimen. However, comparatively, the more efficacious, heterologous prime‐boost technique is also considered a choice of booster regimens in various previous and existing outbreaks, that is, Ebola virus disease (EVD), malaria, tuberculosis, hepatitis B, HIV, and influenza virus. Furthermore, even in the existing pandemic of COVID‐19, many countries such as Germany, Pakistan, Canada, Thailand, and the United Kingdom have started administering the mix‐and‐match technique. Thus, it rationalizes using the available vaccines to assist in the global race to vaccinate the majority to curb the spread of COVID‐19 efficiently.

Besides, there are safety concerns regarding the mix‐and‐match technique due to insignificant large‐controlled trials. Thus, there is no particular guideline or approval from the World Health Organization (WHO) regarding the matter. Therefore, our review aims to advance the understanding of the heterologous prime‐boost immunization strategy relative to a brief positive outlook of trials based on its application in dealing with the outbreaks as mentioned earlier. We also append our article with a set of recommendations for making its revival possible in combatting COVID‐19.

## 2 METHODS

A Boolean search was carried out to find the literature in MEDLINE‐PubMed, Clinicaltrials, and Cochrane Central Register of Controlled Trials databases up till December 22, 2021, using specific keywords that include “SARS‐CoV2,” “COVID‐19,” “Ebola,” “Malaria,” “Tuberculosis,” “Human Immunodeficiency Virus,” “Hepatitis B,” “Influenza,” “Mix and match,” “Heterologous prime‐boost,” with interposition of “OR” and “AND.” The detailed search strategy is provided in the attached Supplementary Material. Full text of all the related articles in English language with Supplementary Appendix was retrieved. In addition, the full text of relevant cross‐references was also retrieved. The articles retrieved are enlisted in Tables 2 and 3. The inclusion criteria for studies were as follows: (a) freely accessible, full articles; (b) original studies, observational and interventional, randomized controlled trials; (c) papers based on the application of heterologous prime‐boost technique for addressing previous and existing outbreaks including COVID‐19; and (d) both published trials in peer‐reviewed journals in English and unpublished trials from clinical trial registries and preprint repositories were included to provide a more general report of serologic data reported to date for the mixed‐dosed approach to add to the literature. However, all the reviews, editorials, commentaries, case reports, case series were excluded.

## 3 DISCUSSION

### 3.1 The mix‐and‐match technique

To begin with, for vaccines to be effective, it usually requires multiple vaccine doses, that is, diphtheria, tetanus, and pertussis (DTP) vaccine. Further, multiple immunizations can be given in either traditional homologous or heterologous prime‐boost format to make the existing vaccines successful. In particular, the concept of the heterologous prime‐boost technique, also known as the mix‐and‐match technique, started budding in the 1990s when HIV researchers tested this strategy. The trials were conducted to analyze the technique because the traditional approach for vaccine delivery was incapable of eliciting the complex immunological mechanisms needed for potential protection from HIV infection.^7^

The strategies practiced so far involve prime dosing of the homologous or the evolving heterologous vaccine regimen followed by a similar or a mix‐and‐match booster, even resulting in a 3‐dose regimen.^8^, ^9^ Regarding the remarkable report of primary outcomes, that is, safety, reactogenicity, and immunogenicity quoted by the pre‐clinical and clinical studies, it is suggestive that the mix‐and‐match procedures employ diverse modes of gene expression that tend to be more immunogenic than the traditional one.^10^

Furthermore, despite the robust immunity offered by it, the mix‐dosing approach against COVID‐19 holds a substantial international interest, as it might mitigate the vaccine supply shocks or shortages as well. Moreover, following the current modifications in guidelines regarding the use of chadox1 to 19 (ChAd) vaccination for COVID‐19 (Vaxzevria, AstraZeneca), several nations are advising to consider the heterologous prime‐boost scheduling of second‐dose alternatives to mRNA vaccines such as Pfizer‐BioNTech (BNT162b2 (BNT) for the vaccinated individuals.^11^ However, more trials are in progress, intending to analyze the use of the mix‐and‐match technique, which will further stimulate an enhanced understanding of the immunological basis of vaccines.

Additionally, the mix‐and‐match technique further outweighs the concept of the existing homologous prime‐dosing regimen based on the general comparison provided in Table 1.

**TABLE 1:** Heterologous prime‐boost technique vs homologous prime‐boost technique (a general comparison)

Advantages	Disadvantages
The mix‐dosing approach provides a wide range of cellular immune responses quantitatively and qualitatively.^12^, ^13^	The heterologous prime‐boost technique may report more short‐term side‐effects, that is, fever and fatigue, than homologous booster after priming with a dose of homologous vaccine.^11^
It can have significant practical applications as in the current global pandemic of COVID‐19 by compensating the scarcity of vaccines supply.^14^	It may also result in worse immune responses in younger individuals as they tend to have a more active immune system than vaccine recipients over the age of 50.^11^
It can expand the scope of immune responses,^15^ resulting in a shift in the immunodominance hierarchy.^16^	
The prime‐boost vaccine approach can also improve the effectiveness of existing vaccines.^17^	
The mixed‐dosing approach further possesses potential in cancer immunotherapy.^16^, ^18^	

### 3.2 An outlook from the history of outbreaks

The heterologous prime‐boost technique, which aims to increase the protective efficacy and rationalize the usage of the available vaccines, can also elicit a more robust and long‐lasting immune response as compared with the single vaccine or traditional homologous prime‐boost immunization regimen. It is made evident by the mixed‐dose approach explicitly adopted in Phase I and II trials quoting excellent safety and improved immunogenicity by the Ebola vaccine developed by Johnson & Johnson.^19^ Notably, the first shot of the Ebola vaccine is composed of the same adenovirus as the AstraZeneca vaccine, and the second shot of an MVA vector (a modified version of a poxvirus: a type that is also under investigation for future Covid‐19 vaccines). Similarly, a pneumococcal conjugate vaccine dose is followed by a booster containing a pneumococcal polysaccharide to trigger more powerful protection in the elderly.^20^ Moreover, according to a study of Kosinska et al, MVA‐HBcore boost vaccination followed by CpG‐application (heterologous prime‐boost regimen) in addressing the outbreak of hepatitis B resulted in a more safe and immunogenic response.^21^

Moving forward, the swift progress of novel vaccination approaches in the past, such as DNA vaccines and viral vector‐based vaccines, has significantly extended the horizon of heterologous prime‐boost vaccination.^22^, ^23^, ^24^ Thus, it is common to use the heterologous prime‐boost approach to address some of the most challenging vaccine development objectives, including tuberculosis, due to the failure of other vaccination approaches. Further, according to a previous study by Magalhaes et al, the core idea was presented to focus on specific crucial antigens and to elicit high‐quality immune responses involving different subsets of T‐cell immune responses, as for the tuberculosis vaccine development, qualitatively and quantitatively different cellular immune responses have been stimulated in rhesus macaques receiving a recombinant Bacille Calmette‐Guerin (BCG) prime followed by an adenovirus 35 vector boost that expressed a fusion protein composed of Ag85A, Ag85B, and TB104.^12^

Alternatively, considering BCG as a boost following a DNA vaccine prime, as in one study conducted in calves, DNA prime with Ag85B, MPT64, and MPT83 antigens followed by a BCG boost was able to elicit higher immune responses and better protection than BCG alone against mycobacterium Bovis challenge.^25^ Additionally, several previous studies also identify the relationship between mix‐and‐match vaccine and age; that is, the beneficial effect is more pronounced when given in early life, as evident in a study about heterologous effects of BCG.^26^ However, another study suggests that early immune response to monovalent influenza vaccine changes significantly after 35 years.^27^

Conclusively, mix‐and‐match approaches generally have been successfully employed in addressing the majority of outbreaks to tackle many others, that is, seasonal influenza virus,^28^, ^29^, ^30^ papillomavirus,^31^ pneumococcus,^32^, ^33^ and rotavirus,^34^ poliovirus,^7^ and a few other examples. Furthermore, though the technique was unsuccessful in combating HIV, it is gaining favor.^16^, ^35^ On the contrary, except for trials NCT01366534^36^ Ad35.CS.01‐RTS, S/AS01 heterologous prime‐boost efficacy against malaria infection is proven unsuccessful as of yet.^37^

Hence, Table 2 summarizes an outlook of, in progress and completed trials aimed at analyzing the efficacy of the heterologous prime‐boost technique in the history of outbreaks, that is, HIV, EVD, malaria, tuberculosis, hepatitis B, and influenza virus, with a well‐referenced summary of results reported so far.

**TABLE 2:** History of outbreaks and the heterologous prime‐boost technique

Outbreaks	Trial ID/study name	Intervention	Results
1. Human immunodeficiency virus (HIV)	NCT02788045^8^	Ad26.Mos.HIV, Ad26.Mos4.HIV	The tetravalent heterologous HIV vaccine regimen is safe and immunogenic than the trivalent heterologous HIV vaccine regimen of Ad26 vaccine.^8^, ^21^, ^38^
2. Ebola virus disease (EVD)	NCT02451891^39^ NCT02485912^40^	Prime vaccine cAd3‐EBO Z followed by the boost vaccine, MVA EBO Z	This Phase I trial in humans proved it safe and immunogenic and encourages further testing in phase 2 and 3 studies. The 1‐week prime‐boost interval regimen appeared to be particularly suitable for outbreak control.^41^
	NCT02313077^42^	Primary immunization with Ad26.ZEBOV; boosting by MVA‐BN‐Filo	Primary vaccination with Ad26.ZEBOV; boosting by MVA‐BN‐Filo resulted in a sustained increase in specific immunity.^43^, ^44^ Moreover, further assessment of these vaccines in phase 2^45^ and three studies^46^ is ongoing.
3. Malaria	NCT01366534^36^	Ad35.CS.01‐RTS,S/AS01	Results showed unremarkable efficacy.^37^
4. Mycobacterium tuberculosis	^47^	BCG Vaccine dosing boosted both by SeV85AB prime‐DNA boost (SeV85AB‐DNA) and DNA prime‐SeV85AB boost (DNA‐SeV85AB) vaccination strategies	A heterologous prime‐boost regimen with a novel recombinant SeV85AB and a DNA vaccine increases the immunity above those from a single vaccine, indicative of higher T cells produced in response to it.^47^
	^48^, ^49^, ^50^	BCG Vaccine boosted with MVA85A.	BCG‐MVA85A induced a significant immunogenic response compared to BCG dosing alone.
5. Hepatitis B	^21^	MVA‐HBcore boost vaccination followed by CpG‐application	Results indicated a more potent HBV‐specific CD8 T‐cell immunity that enhanced control of hepatocytes replicating HBV.^21^
6. Influenza	NCT00841763^28^	Trivalent influenza virus vaccine (TIV), adjuvanted monovalent influenza virus vaccine (aH5N1), adjuvanted trivalent influenza virus vaccine (aTIV)	Adequate immunity levels induced by the two doses of the MF59‐H5N1 vaccine are contrary to homologous and cross‐clade A/H5N1 virus. Thus, it supports the appropriateness of the MF59‐adjuvanted A/H5N1 vaccine.^29^

### 3.3 Researchers and the battle of COVID‐19

The decades‐long‐struggling in the HIV vaccine field has driven scientists to develop innovative vaccine platforms, including DNA, mRNA, and viral vectors such as the adenovirus, which enabled the quicker development of COVID‐19 vaccines.^7^ Further, the proven efficacy results from the deployment of the heterologous prime‐boost technique in clinical trials addressing the previous and existing outbreaks have promoted considering the heterologous prime‐boost approach in combatting COVID‐19, as discussed below.

The efforts to prove the efficacy of this regimen in battling the COVID‐19 consisted of Phase 1/2 trials comprising combinations of seven mix‐and‐match vaccines, including Moderna, Pfizer, AstraZeneca, Johnson & Johnson, Sinopharm, Sinovac, and Adenovirus‐vectored vaccine. The average patient population in these trials lay between 26 and 3000. The primary determining outcome was antibody titers against SARS‐COV protein two after administering these heterogeneous shots to assess the potency of this method. These assessments included levels of anti‐spike immunoglobulins using ELISA at baseline and 4 weeks post‐boost, and many others. (ISRCTN69254139, UK) An electronic diary card also measured incidences of serious adverse events.

However, the few limitations of the process mentioned above included exceptional disruption in vaccine distribution. People could not get the second dose after their first one, and some vaccines were unavailable for emergency use. In contrast, some vaccines might not have the same technological platform, so mixing was impossible. Further, not enough trials are available to prove anything as of yet.^20^ Therefore, since it is unchartered territory, governments should be meticulous in keeping control of only mixing vaccines in trial studies and not in the general public due to a lack of evidence for the established efficacy of these trials.

Furthermore, there should be a plan to conduct more studies, given that it is struggling for developing or under‐developed countries to keep up with the mass production of certain vaccines. Remarkably, considering the preprint data will favor the progress and decrease the disease burden. Governments of countries such as Germany, Canada, etc,^51^ where such trials are already thriving, should spread awareness across the globe and inform the unlearned.

The following vaccines have been experimented with in a heterologous schedule in some cohorts: Moderna and Pfizer, AstraZeneca and Moderna, AstraZeneca and Pfizer. Moderna and Pfizer are RNA vaccines, while AstraZeneca is a viral vector vaccine. Administration of the said vaccines after required periods can help achieve higher antibody titers and T‐cell responses^52^ in populations and eventually decrease the burden on mass production of certain vaccines. All of this is excellent news for immunocompromised patients with lower CD cell counts.

Subsequently, Table 3 summarizes the details of discussed and further clinical trials, incorporating COVID‐19 vaccines that are currently in progress for evaluating the efficacy of the technique.

**TABLE 3:** Details of ongoing studies on COVID‐19 for testing the efficacy of the heterologous prime‐boost technique

S.No. trial ID/study title/author	Study design	Location	Intervention	Study status	Findings
1. MOSAIC; NCT04894435^52^	Interventional (Clinical Trial) n = 1300	Canada	Moderna, Pfizer, AstraZeneca	Recruiting start date: 20/05/21 End Date: 03/23	N/A
2. NCT04889209^53^	Interventional (Clinical Trial) n = 950	USA	Moderna, Pfizer, Johnson & Johnson	Recruiting Start Date: 28/05/21 End Date: 01/12/22	In comparison to homologous boost, which increased neutralizing antibody titers from 4.2 to 20‐fold, the heterologous boost increased titers to a more excellent value from 6.2 to 76‐fold^54^
3. NCT04998240^55^	Interventional (Clinical Trial) n = 360	Mozambique	Sinopharm and AstraZeneca	Not yet recruiting Start Date: 01/09/21 End Date: 30/10/22	N/A
4. CombiVacS; NCT04860739^56^	Interventional (Clinical Trial) n = 676	Spain	Pfizer and AstraZeneca	Active, not recruiting Start Date: 24/04/21 End Date: 30/04/22	No report of severe adverse effects. Thus, participants primed with ChAdOx1‐S and boosted with BNT162b2 induced a more potent immunogenic response in comparison to the control group (no vaccination/ on observation)^57^
5. Groß R. et. al^7^	Cohort Study n = 26	Germany	AstraZeneca prime and Pfizer boost	Ongoing	The Heterologous ChAdOx1 nCoV‐19 / BNT162b2 prime‐boost regimen induces a more potent immunogenic response as indicated by a significant rise in antibody titers compared to homologous vaccination.^7^
6. EudraCT‐No. 2021‐001512‐28^58^	Prospective, Observational Cohort Study n = 340	Germany	Pfizer and AstraZeneca	Ongoing, date for study entry: 13/04/21	N/A
7.ISRCTN69254139^11^	Primary: Multi‐center single‐blind phase II Secondary: Randomized Parallel Study n = 830	UK	Pfizer and AstraZeneca	Ongoing date applied: 29/01/21	N/A
8. NCT04892459^59^	Clinical Trial n = 300	China	Sinopharm and adenovirus‐vectored vaccine Inactive SARS‐CoV‐2 vaccine (Vero cell) and Recombinant SARS‐CoV‐2 Ad5 vectored vaccine booster	Active, not recruiting Start Date: 25/05/21 End Date: 25/12/21	N/A
9. ARNCOMBI; NCT04900467^60^	Interventional (Clinical Trial) n = 418	France	Pfizer and Moderna (Homologous vs Heterologous vaccine regimen)	Active, not recruiting Start Date: 28/05/21 End Date: 01/22	N/A
10. HeVac; NCT04907331^61^	Interventional (Clinical Trial) n = 3000	Austria	Pfizer and Astrazeneca (Vaxzevria and Comirnaty)	Recruiting Start Date: 10/05/21 End Date: 30/12/21	N/A
11. NCT05049226^62^	Interventional (Clinical Trial) n = 1320	Thailand	Sinovac and AstraZeneca or Pfizer (booster)	Not yet recruiting Start Date: 24/09/21 End Date: 09/23	N/A
12. NCT04760730^63^	Interventional (Clinical Trial) n = 100	United Arab Emirates	AZD1222 and rAd26‐S one after other interchangeably (to analyze heterologous prime‐boost use of either)	Recruiting Start Date: 13/07/21 End Date: 30/06/22	N/A
13. NCT05079633^64^	Interventional (Clinical Trial) n = 220	Taiwan	Moderna, Medigen COVID‐19 Vaccines Homologous boost schedule (first dose mRNA 1273, second dose mRNA 1273) Heterologous boost schedule (first dose mRNA 1273, second dose MVC COV1901)	Active, not recruiting Start Date: 30/09/21 End Date: 06/22	N/A
14. [PRIBIVAC] NCT05142319^65^	Interventional (Clinical Trial) n = 600	Singapore	Moderna, Pfizer (mRNA booster) & non‐mRNA booster vaccine (A,B,C) (Homologous boost regimen (control group) vs Heterologous boost regimens (intervention group))	Recruiting Start Date: 12/10/21 End Date: 04/23	N/A
15. NCT05132855^9^	Interventional (Clinical Trial) n = 400	Taiwan	BNT162b2 (Pfizer), mRNA‐1273 (Moderna), MCV COVID‐19 vaccine (Medigen) (3 dose regimen)	Recruiting Start Date: 30/11/21 End Date: 04/23	N/A

### 3.4 Challenges: The grey area

More importantly, many previous studies have postulated that this approach of mix‐and‐match results in much better protection with an increase in antibody titer compared with those with homologous or the same vaccine approach (Tables 2 and 3) to some extent. On the flip side, the reported incidence of some adverse effects in ongoing and completed trials on analyzing the technique's potency in the history of outbreaks underscores the doubt in its appropriateness in the battle of COVID‐19. Concerning the previous studies that have pointed out that after the mix‐and‐match approach of monovalent HINI influenza vaccine, there was an increased incidence of narcolepsy in adolescents and children, with the incidence decreasing with the increase in age as the subjects approached adult life.^66^, ^67^, ^68^ Additionally, according to the study by Shaw et al, the mix‐and‐match technique results in higher reactogenicity, reporting worse immune responses in young vaccine recipients compared with individuals over 50 years of age. Thus, it creates an atmosphere of mistrust regarding heterologous vaccination as well.^11^ Furthermore, there are logistical barriers, that is, a small proportion of volunteers showing up for the trials due to safety concerns regarding the technique. As a result, there is an insignificant number of large‐controlled trials conducted to test a mixed‐dose approach for COVID‐19 vaccines.

In short, where the mix‐dose approach elicits robust immunogenicity and tolerable reactogenicity on administration, on the other side favor and suggest only the mix over no second dose.^69^, ^70^ Further, according to one of the chief scientists at WHO, this mix‐and‐match approach may lead to “a chaotic situation.”^52^ Consequently, predominantly due to the limitations mentioned earlier, the safety and efficacy of the heterologous prime‐boost technique, in general, remains a grey area. Hence, there is no particular guideline or approval from the World Health Organization (WHO) regarding the matter so far.

## 4 RECOMMENDATION AND THE FUTURE INSIGHTS

The heterologous prime‐boost technique is not something novel as provided by the outlook of trials from the history of outbreaks earlier (Table 2) and comparatively proved to be a more effective technique than a homologous one. The mix‐and‐match approach also compensates for the scarce vaccine supplies and unequal roll‐out of COVID‐19 vaccines. Therefore, it paves the way for the method to be further trialed in existing COVID‐19 vaccines (proven safe by WHO) on an immediate and large‐scale basis.

However, due to the safety concerns, the sample size for trials is an area of concern. Thus, incentives such as awareness campaigns and commercials focusing not only on clearing the mistrust regarding safety concerns of the technique but highlighting the long‐lasting immunologic benefits from getting the mix‐dosed booster jab would encourage the attention of more volunteers. Subsequently, in the long run, the considerable sample size of the trials will provide strong evidence to support the relevance of the heterologous prime‐boost technique. Further, considering parameters like pharmaceutical aspects of vaccines and clinical aspects and high demographic variance while conducting trials would aid in providing a more comprehensive analysis of the reactogenicity of the technique.^11^, ^25^ Moreover, making the availability of more preprint data can make meta‐analyses possible, which would also aid in overcoming the barrier of sample size.

Consequently, with the whole world getting on the same page by prioritizing the technique to vaccinate their citizens, it would act as a more prompt attempt than other preventive measures (ie, wearing masks and strict lock‐downs) to curtail the pace of mutation of COVID‐19 effectively and efficiently. Additionally, we can further expand the scope of immune responses by addressing the current scenario timely with serious consideration of reviving the heterologous prime‐boost technique.^15^ Thus, eventually can prepare researchers to devise countermeasures ahead of time to combat other priority diseases^71^ coined by WHO, that is, “Disease X” in 2018 and different much challenging variants of concerns, including Omicron, competently.

## 5 CONCLUSION

Conclusively, the mix‐and‐match technique provides more long‐lasting immunological and logistical benefits than the traditional one. Additionally, providing a brief outlook in dealing with previous and existing outbreaks in the article promotes the revival of the technique in combatting the COVID‐19. However, further studies evaluating the strategy incorporating vaccines, such as Moderna, Pfizer, AstraZeneca, and Covax, are ongoing and are also crucial to informing the appropriateness of heterologous prime‐boost schedules. Consequently, the future insight, after approval of this technique across the world, would result in healthcare policy makers to competently preclude the evolution of other priority outbreaks according to WHO, in turning into the “next pandemic” or “next public health emergency” to eventually eradicate the risk of “population crisis.”

## FUNDING

None declared.

## CONFLICT OF INTEREST

The authors declare no conflicts of interest.

## AUTHOR CONTRIBUTIONS

Conceptualization: Amna Siddiqui, Alishba Adnan, Munib Abbas, Shafaq Taseen, Sidhant Ochani, Mohammad Yasir Essar.

Methodology: Amna Siddiqui, Alishba Adnan, Munib Abbas, Shafaq Taseen.

Project Administration: Amna Siddiqui.

Supervision: Amna Siddiqui.

Visualization: Amna Siddiqui, Alishba Adnan, Sidhant Ochani.

Writing—Original Draft: Amna Siddiqui, Alishba Adnan, Munib Abbas, Shafaq Taseen, Sidhant Ochani, Mohammad Yasir Essar.

Writing—Review and Editing: Amna Siddiqui, Alishba Adnan, Munib Abbas, Shafaq Taseen, Sidhant Ochani, Mohammad Yasir Essar.

Author Approval: None to declare.

Data Integrity: None to declare.

## Supporting information


**Data S1.** Supporting information.
