# Supplementary material for: Revival of the heterologous prime‐boost technique in COVID‐19: An outlook from the history of outbreaks
Source: Health Sci Rep. 2022 Feb 23;5(2):e531. doi: 10.1002/hsr2.531 (PMC8866911; doi:10.1002/hsr2.531)
Supplement: Supplementary file 1 — Data S1. Supporting information. [file HSR2-5-e531-s001.docx]

| Search details For Table 2: History of Outbreaks and the Heterologous Prime Boost Technique (PubMed and ClinicalTrials) | |
| --- | --- |
| 1. Human Immunodeficiency Virus (HIV) | Human Immunodeficiency Virus AND Heterologous Prime Boost OR Mix and Match |
| 2. Ebola Virus Disease (EVD) | Ebola Virus Disease AND Heterologous Prime Boost OR Mix and Match |
| 3. Malaria | Malaria AND Heterologous Prime Boost OR Mix and Match |
| 4. Mycobacterium Tuberculosis | Tuberculosis AND Heterologous Prime Boost OR Mix and Match |
| 5. Hepatitis B | Hepatitis B AND Heterologous Prime Boost OR Mix and Match |
| 6. Influenza Virus | Influenza Virus AND Heterologous Prime Boost OR Mix and Match |
|  |  |
| Search Details for Table 3: Details of ongoing studies on COVID-19 for testing the efficacy of the Heterologous Prime Boost technique | |
| 1. PubMed | (("SARS-CoV2"[All Fields] OR ("covid 19"[All Fields] OR "covid 19"[MeSH Terms] OR "covid 19 vaccines"[All Fields] OR "covid 19 vaccines"[MeSH Terms] OR "covid 19 serotherapy"[All Fields] OR "covid 19 serotherapy"[Supplementary Concept] OR "covid 19 nucleic acid testing"[All Fields] OR "covid 19 nucleic acid testing"[MeSH Terms] OR "covid 19 serological testing"[All Fields] OR "covid 19 serological testing"[MeSH Terms] OR "covid 19 testing"[All Fields] OR "covid 19 testing"[MeSH Terms] OR "sars cov 2"[All Fields] OR "sars cov 2"[MeSH Terms] OR "severe acute respiratory syndrome coronavirus 2"[All Fields] OR "ncov"[All Fields] OR "2019 ncov"[All Fields] OR (("coronavirus"[MeSH Terms] OR "coronavirus"[All Fields] OR "cov"[All Fields]) AND 2019/11/01:3000/12/31[Date - Publication]))) AND ("Mix"[All Fields] AND ("match"[All Fields] OR "matched"[All Fields] OR "matches"[All Fields] OR "matching"[All Fields] OR "matchings"[All Fields]))) OR (("heterologous"[All Fields] OR "heterologously"[All Fields]) AND "prime-boost"[All Fields]) |
| 2. ClinicalTrials | SARS-COV-2 OR COVID-19 AND Heterologous Prime Boost OR Mix and Match |

**SUPPLEMENTARY MATERIAL**
